# Spatial transcriptomic analysis of virtual prostate biopsy reveals confounding effect of tissue heterogeneity on genomic signatures

**DOI:** 10.1186/s12943-023-01863-2

**Published:** 2023-10-03

**Authors:** Sandy Figiel, Wencheng Yin, Dimitrios Doultsinos, Andrew Erickson, Ninu Poulose, Reema Singh, Anette Magnussen, Thineskrishna Anbarasan, Renuka Teague, Mengxiao He, Joakim Lundeberg, Massimo Loda, Clare Verrill, Richard Colling, Pelvender S. Gill, Richard J. Bryant, Freddie C. Hamdy, Dan J. Woodcock, Ian G. Mills, Olivier Cussenot, Alastair D. Lamb

**Affiliations:** 1 Nuffield Department of Surgical Sciences, University of Oxford, Old Road Campus Research Building, Oxford, OX3 7DQ UK; 2 Department of Urology, Oxford University Hospitals NHS Foundation Trust, Oxford, UK; 3 Department of Cellular Pathology, Oxford University Hospitals NHS Foundation Trust, Oxford, UK; 4 Science for Life Laboratory, Department of Gene Technology, KTH Royal Institute of Technology, Solna, Sweden; 5 Department of Pathology and Laboratory Medicine, Weill Cornell Medicine, New York, NY USA; 6 Oxford NIHR Biomedical Research Centre, Oxford University Hospitals NHS Foundation Trust, Oxford, UK

**Keywords:** Prostate cancer, Virtual biopsy, Prognostic genetic signatures, Spatial transcriptomics

## Abstract

Genetic signatures have added a molecular dimension to prognostics and therapeutic decision-making. However, tumour heterogeneity in prostate cancer and current sampling methods could confound accurate assessment. Based on previously published spatial transcriptomic data from multifocal prostate cancer, we created virtual biopsy models that mimic conventional biopsy placement and core size. We then analysed the gene expression of different prognostic signatures (OncotypeDx®, Decipher®, Prostadiag®) using a step-wise approach with increasing resolution from pseudo-bulk analysis of the whole biopsy, to differentiation by tissue subtype (benign, stroma, tumour), followed by distinct tumour grade and finally clonal resolution. The gene expression profile of virtual tumour biopsies revealed clear differences between grade groups and tumour clones, compared to a benign control, which were not reflected in bulk analyses. This suggests that bulk analyses of whole biopsies or tumour-only areas, as used in clinical practice, may provide an inaccurate assessment of gene profiles. The type of tissue, the grade of the tumour and the clonal composition all influence the gene expression in a biopsy. Clinical decision making based on biopsy genomics should be made with caution while we await more precise targeting and cost-effective spatial analyses.

**Supplementary Information:**

The online version contains supplementary material available at 10.1186/s12943-023-01863-2.

Prostate cancer (PCa) progression is unpredictable. Some tumours are indolent, whilst others tumours are aggressive with rapid progression and metastasis [1]. Recent prognostic genetic signatures have added a molecular dimension to therapeutic decision-making [2, 3]. However, a drawback of biopsy-derived molecular signatures is inaccurate sampling and failure to properly consider intra-tumoral heterogeneity [2, 4, 5]. Inadequate sampling of multiclonality in prostate cancer can lead to a misleading assessment of tumour heterogeneity, potentially resulting in suboptimal treatment decisions. Failure to account for diverse clonal populations may hinder the identification of aggressive subclones and the development of more effective therapeutic strategies. In light of these challenges, we sought to investigate the impact of tumour heterogeneity on prognostic genomic signatures, using the unique spatial resolution of transcriptomic and inferred genomic information generated in our previous work [6]. We compared three different prognostic signatures (OncotypeDx® [7], Decipher® [8] and ProstaDiag® [9]) in virtual biopsy models using spatial transcriptomics (ST). These signatures include genes associated with biological processes that may be fundamental to tumour development. In evaluating these signatures, we assessed gene expression assigned to these consensus processes.

Gene expression analysis was performed on radical prostatectomy tissue from a patient with multifocal PCa. We used our recently published organ-wide ST data [6] to construct virtual biopsy models that mimic conventional biopsy placement and core size (Fig. 1). The biopsy regions were intentionally positioned to represent maximum heterogeneity. All spots containing less than 500 Unique Molecular Identifier counts were removed. Genes detectable at this threshold in less than 10% of spots were also removed. Overall, we examined the expression of 9 of the 12 OncotypeDx® genes, 11/19 Decipher® genes, and 28/36 Prostadiag® genes, excluding housekeeping genes. We extracted gene expression data from the barcode ID of designed biopsies (LoupeBrowser, v6.3.0) using R (v4.22). In all analyses, we normalised the libraries using *spaceranger aggr* (v2.0). Fold changes with false discovery rate were analysed using *EdgeR* (v3.40.1).

**Fig. 1:**
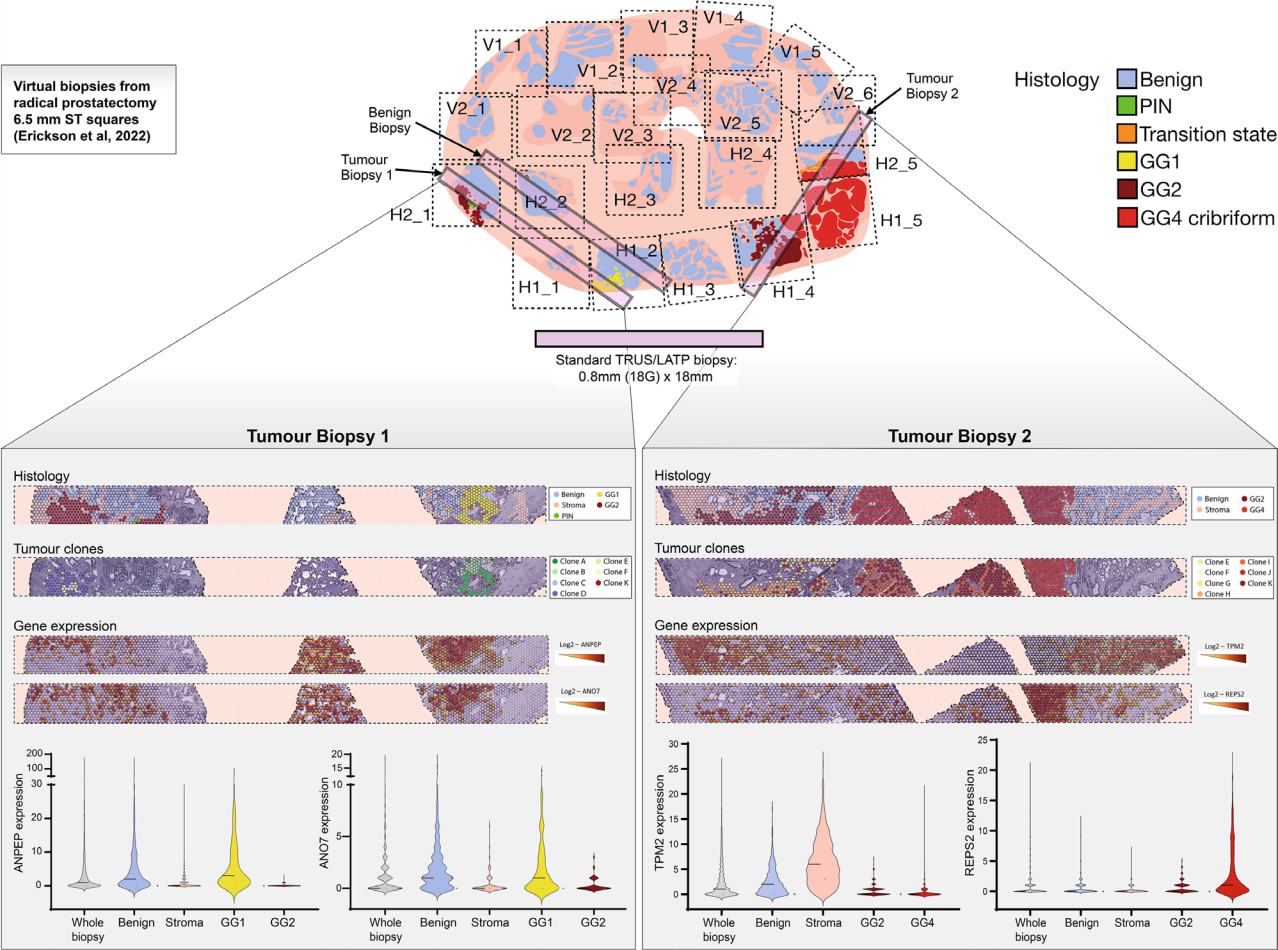
Spatial visualisation of virtual biopsies. We used our recently published organ-wide spatial transcriptomic data [9] to construct virtual biopsy models (2 tumour biopsies and 1 benign biopsy) that mimic conventional biopsy placement and core size. Visualisation of histology and tissue status (GG: Gleason grade group; PIN: prostatic intraepithelial neoplasia) and tumour clones from each tumour biopsy. Spatial visualisation of gene expression (ANPEP, ANO7, TPM2 and REPS2) in each tumour biopsy. Violin plots representing gene expression according to histological status. TRUS = transrectal ultrasound guided prostate biospy. LATP = local anaesthetic transperineal prostate biospy. ST = spatial transcriptomics. GG = Gleason grade group. PIN = prostatic intra-epithelial neoplasia. 18G = 18 gauge core biopsy needle

We compared the gene expression of virtual tumour biopsies to a benign control at increasing levels of resolution: from whole biopsy, through tissue subtype (benign, stroma, tumour) and tumour grade, to clonal level (Fig. 1). A consensus pathology underpins this analysis, with two pathologists independently annotating each 55 μm ST spot (approximately 10–15 cells).

We observed clear evidence of variation in the expression of constituent genes from different prognostic signatures within biopsies (Fig. 2). In tumour biopsy 1, the expression of the cellular organisation markers (FLNC, TPM2, GSN) of OncotypeDx® signature was decreased in a Gleason Grade Group (GG) 1 area (logFC=-0.91; -1.05; -0.68 respectively), while it was increased in the region of GG2 cancer (logFC = 0.73; 1.09 respectively), compared to the control biopsy. Importantly, this distinction would be lost if either the whole biopsy, or tumour areas alone, were analysed in bulk (Fig. 2A). We observed similar results when we extended these analyses to other prognostic signatures (Supplementary Fig. 1A & 2). Further differences in gene expression were found between GG1 and GG2 cells compared to the control, such as the expression of genes involved in epithelial-mesenchymal transition (ANPEP, COL1A1, COL1A2, FMOD, SPARC), transport (ANO7, CHRNA2) and cell cycle (NCAPD3, ZWILCH).

**Fig. 2:**
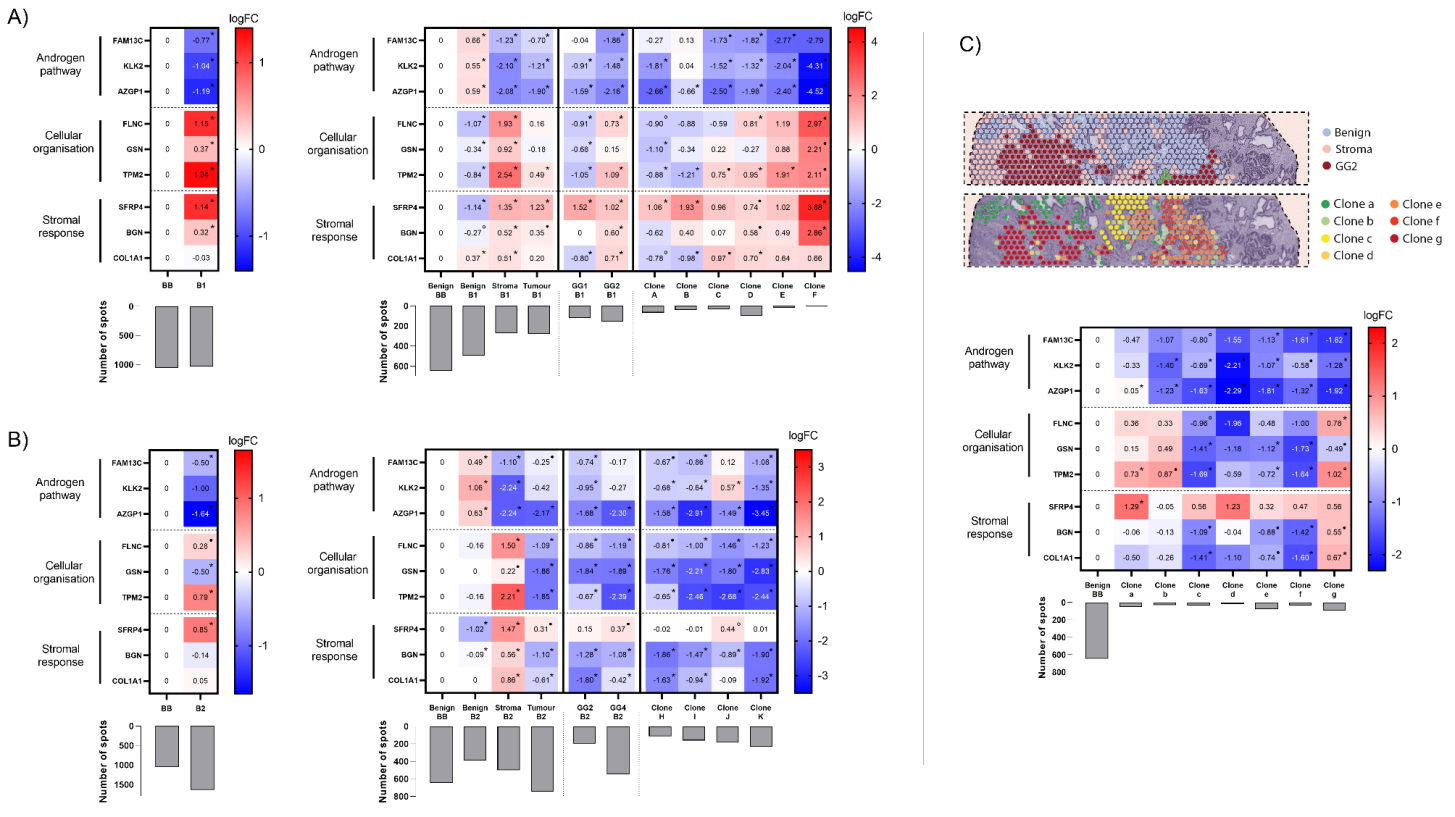
Gene expression profile of the OncotypeDx® signature. Heatmap of gene expression (logFC) of the OncotypeDx® signature at different levels of precision (whole biopsy, tissue subtype, tumour grade and clonal level) in tumour biopsy 1 (**A**), tumour biopsy 2 (**B**) and section H2_1 (**C**). The histograms represent the number of spatial transcriptomic spots for each entity. False discovery rate (FDR) is indicated: *FDR < 0.01; ^•^FDR < 0.05, °FDR < 0.1. FC: fold change; BB: benign biopsy; B1: tumour biopsy 1; B2: tumour biopsy 2; GG: Gleason grade group

Previously [6], we interrogated the expression data using spatial inferred copy number variations (siCNV) and constructed a phylogenetic-tree to describe sequential clonal events in tumour regions of the tissue. We therefore performed clonal-level analyses based on the previously identified 11 clonal groups of tumour cells (clone A to clone K) [6]. We found expression differences between clones of the same histological grade, further supporting the need to respect heterogeneity in interpreting genomic scores (Fig. 2A; Supplementary Fig. 1A & 2).

In tumour biopsy 2, the expression of the cellular organisation markers (FLNC, TPM2) and the stromal response markers (BGN, COL1A1) decreased in GG2/GG4 areas compared to the benign control (logFC = -0.86/-1.14; -0.62/-2.39; -1.28/-1.08; -1.80/-0.42 respectively). Conversely, these genes demonstrated an inverse profile if the biopsy was analysed in bulk (logFC = 0.28; 0.79; -0.14; 0.05) (Fig. 2B). Therefore, the overall gene expression profile of biopsy 2 as a whole, which comprises 46% tumour tissue and 31% stroma, did not reflect the gene expression profile of the tumour areas. this is because the tumour profile was masked by the stroma profile. In addition, the expression of the androgen pathway markers (FAM13C, KLK2) of OncotypeDx® were decreased in a region of GG2 cancer (logFC = -0.74; -0.95 respectively) but not in the GG4 area (logFC = -0.17; -0.27 respectively) (Fig. 2B**).** Similar to Biopsy 1, this information would be missed if either the whole biopsy, or tumour areas alone, were analysed in bulk. We obtained consistent results when we extended these analyses to the other genomic scores. As with biopsy 1, we also observed differences between tumour clones (Supplementary Fig. 1B & 3).

Given our previous finding of early cancer-associated events in histo-pathologically benign prostate tissue (section H2_1) [6], we proceeded to a detailed examination of this region (Fig. 2C; Supplementary Fig. 4). We interrogated the expression of the different signatures in the 7 clonal groups previously identified: clones a and c comprise 100% benign cells; clones d to f 25% and clone g less than 25%. The gene expression profile of clone c, previously identified as ‘altered benign’, was closer to that of tumour clones d, e and f than to that of benign clones a and b, in the 3 prognostic signatures studied. For the OncotypeDx® signature, markers of cell organisation (FLNC, GSN, TPM2) and stromal response (BGN and COL1A1) decreased in clone c cells (logFC = -0.96; -1.41; -1.69; -1.09; -1.41 respectively), in clear distinction from clones a/b (logFC = 0.36/0.33; 0.15/0.49; 0.73/0.87; -0.06/-0.13; -0.50/-0.26 respectively) compared to the benign control. These interesting findings from an area of altered benign tissue suggest an intermediate state between benign and malignant cells, which would not have been identified in a bulk analysis.

The effectiveness of prognostic genomic tests depends on accurately deciphering the genomic landscape of a heterogeneous and unpredictable cancer for each patient [3, 10]. Inaccurate measurement of genomic composition can lead to an incorrect assessment of the patient’s risk level and, consequently, the implementation of an ineffective treatment plan.

We are not the first to highlight this problem. Other studies have reported the confounding effects of heterogeneity in genomic tests [2, 11, 12]. By interrogating these issues in a spatial dimension, we can now provide a detailed rationale for the shortcomings of current genomic techniques. Despite their high cost, we believe that ST analyses can provide a comprehensive in situ transcriptional assessment of heterogeneity. Until we advance this understanding alongside more accurate targeting methods and cost-effective spatial analyses, we recommend caution when making clinical decisions based on genomic analysis of prostate biopsies.

We recognise the limitations of presenting data from a single patient. We also point out that there are other examples of molecular heterogeneity (e.g. point mutations and epigenetic changes) that are not captured by the ST approach. It will be interesting to replicate these analyses as we put further prostates through our organ-scale ST pipeline. Longitudinal sampling will also be valuable to inform clonal development over time. The current cost for organ-scale analysis of a single prostate is around £100,000, but as such costs fall, we anticipate developing a more comprehensive understanding of PCa heterogeneity and lethal clonality. Furthermore, the accessibility of insights derived from these spatial molecular technologies will likely improve with the integration of machine learning into morpho-molecular studies, paving the way for a new era of precision medicine.

In conclusion, in this short article we show that tissue type, tumour grade, and clonal composition of tissue all influence gene expression within a biopsy sample. Bulk analyses of prostate biopsies for genomic scores used in clinical practice effectively ignore these distinctions. To maximise the potential of biopsy-based genomics in clinical decision-making, we believe that precise targeting will need to be combined with more granular spatial analyses, in order to provide accurate scores which will better-inform clinical decisions.

### Electronic supplementary material

Below is the link to the electronic supplementary material.

Supplementary Material 1

Supplementary Material 2

Supplementary Material 3

Supplementary Material 4

## Data Availability

The data that support the findings of this study are available from the European Genome-phenome Archive repository, https://ega-archive.org/studies/EGAS00001006124. Sequencing data for the prostate sample have been deposited at the European Genome-phenome Archive (EGA; www.ebi.ac.uk/ega/), which is hosted by the European Bioinformatics Institute (EBI), under accession number EGAS00001006124. The data are available under Data Use Conditions (DUO) and are limited to not-for-profit use as well as health/medical/ biomedical purposes. Access is granted if the above criteria are fulfilled, and local institutional review board/ethical review board approvals are provided. High resolution histological images, count matrices and other additional material are available on Mendeley Data (https://doi.org/10.17632/svw96g68dv.4).

## Abbreviations

B1: tumour biopsy 1
B2: tumour biopsy 2
BB: benign biopsy
FC: fold change
FDR: False discovery rate
GG: Gleason Grade Group
PCa: prostate cancer
ST: spatial transcriptomics
