# Supplementary figures and images for: Spatial transcriptomic analysis of virtual prostate biopsy reveals confounding effect of tissue heterogeneity on genomic signatures
Source: Mol Cancer. 2023 Oct 3;22:162. doi: 10.1186/s12943-023-01863-2 (PMC10546768; doi:10.1186/s12943-023-01863-2)

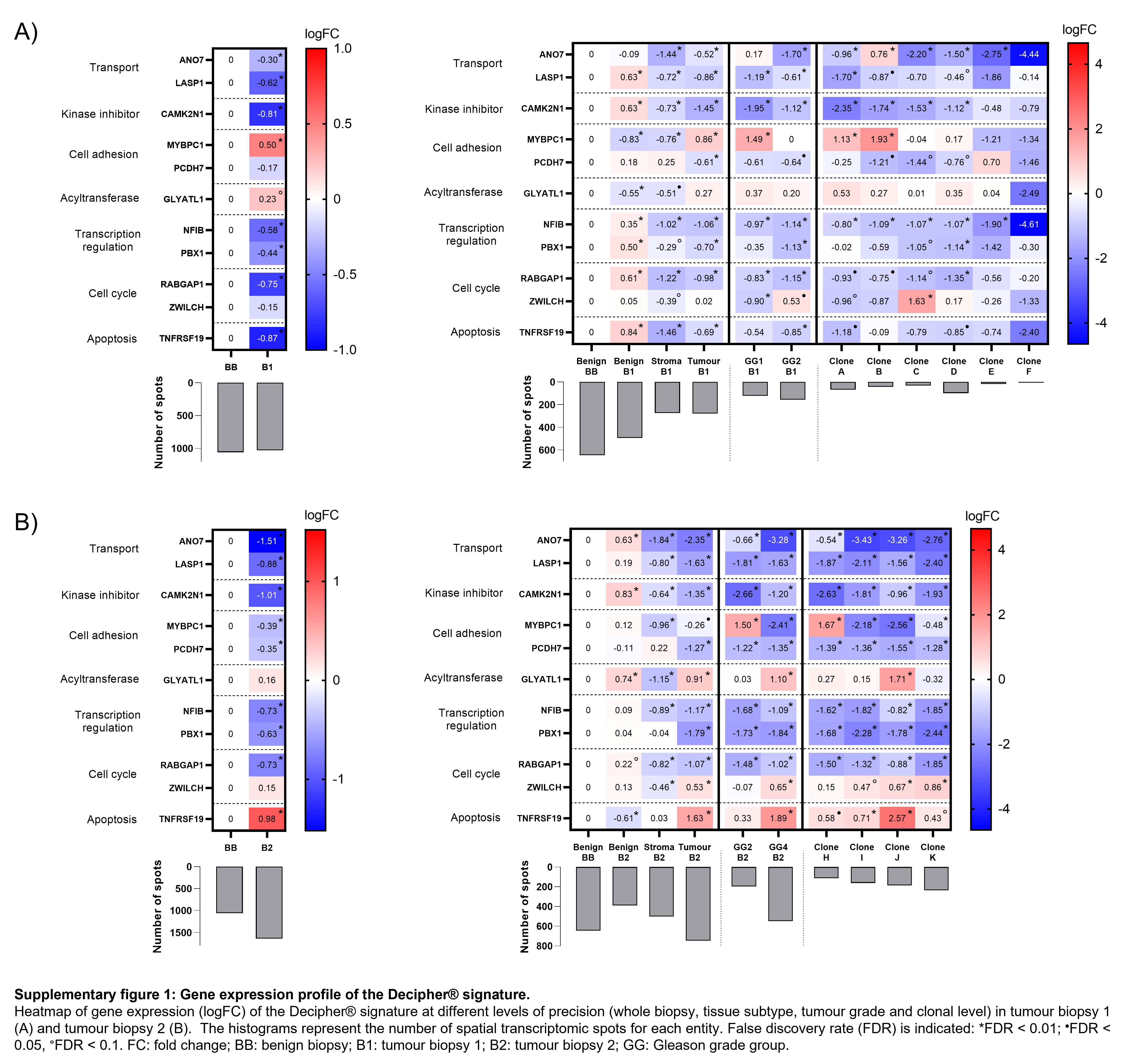

Supplement: Supplementary file 1 — Supplementary Material 1 [file 12943_2023_1863_MOESM1_ESM.png]

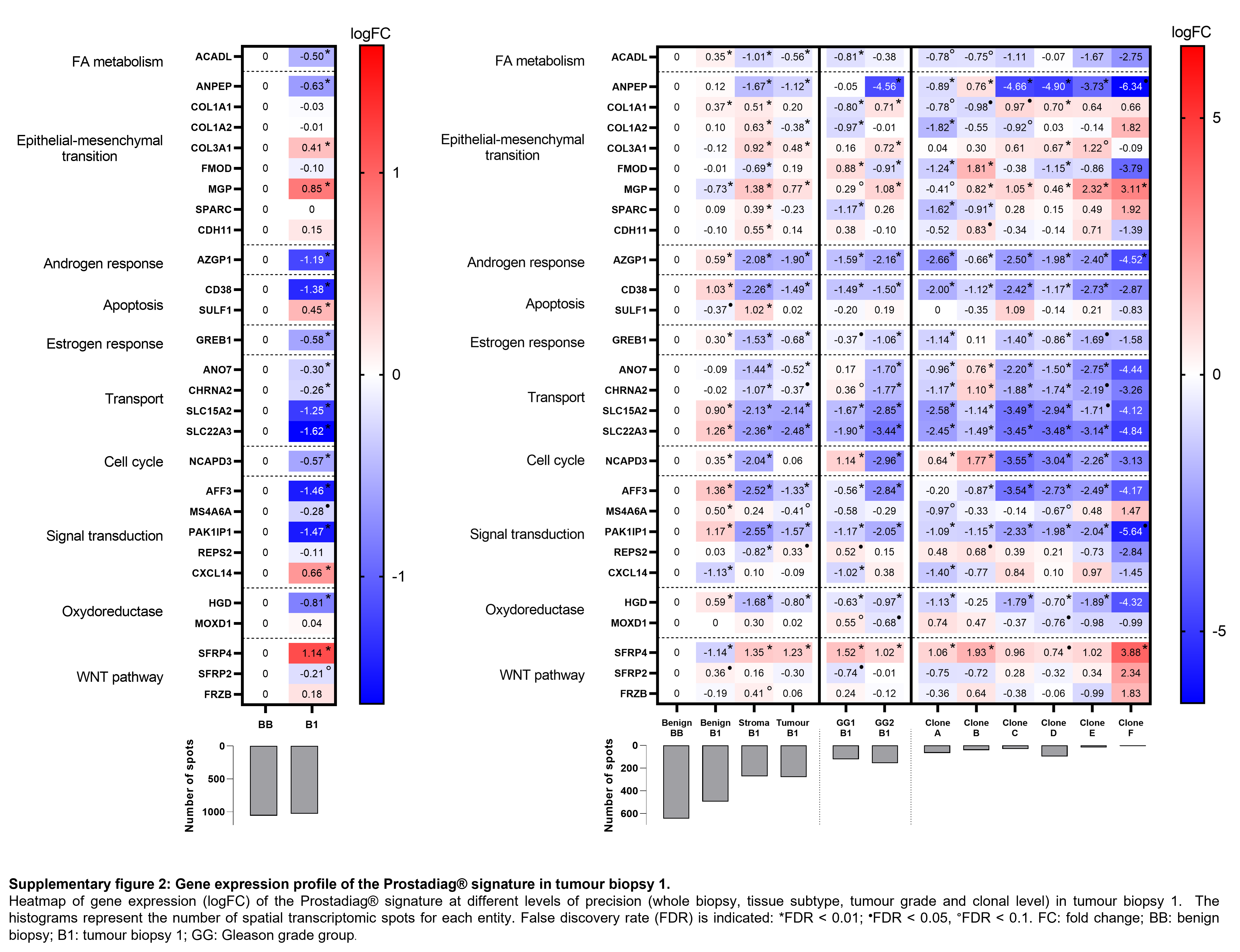

Supplement: Supplementary file 2 — Supplementary Material 2 [file 12943_2023_1863_MOESM2_ESM.png]

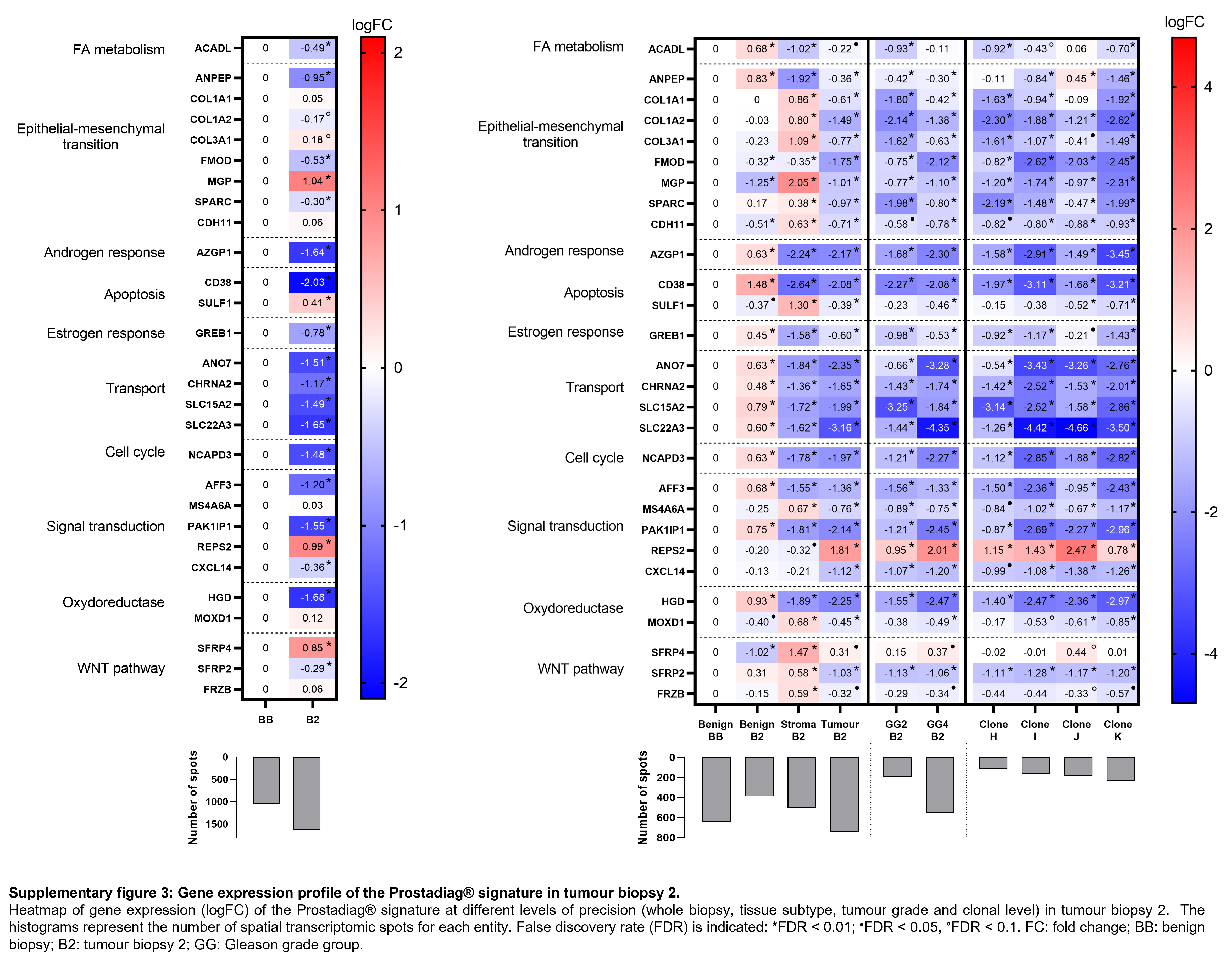

Supplement: Supplementary file 3 — Supplementary Material 3 [file 12943_2023_1863_MOESM3_ESM.png]

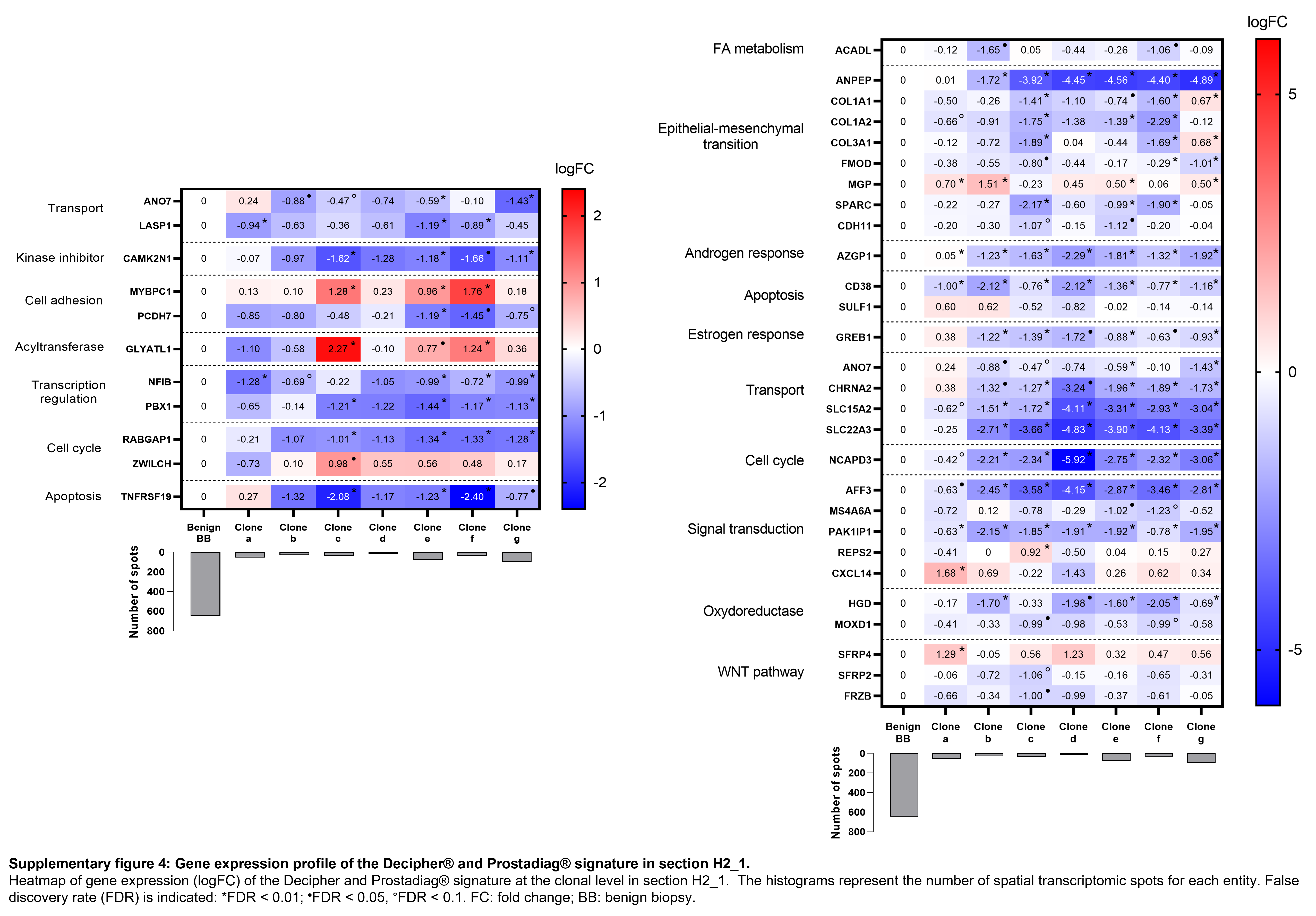

Supplement: Supplementary file 4 — Supplementary Material 4 [file 12943_2023_1863_MOESM4_ESM.png]
